# In the shadow of bad news – views of patients with acute leukaemia, myeloma or lung cancer about information, from diagnosis to cure or death

**DOI:** 10.1186/1472-684X-6-1

**Published:** 2007-01-24

**Authors:** Lena Hoff, Ulf Tidefelt, Lars Thaning, Göran Hermerén

**Affiliations:** 1 Department of Medical Ethics, Lund University, Sweden; 2 Department of Medicine, Örebro University Hospital, Sweden; 3 Department of Pulmonary Medicine, Örebro University Hospital, Sweden

## Abstract

**Background:**

Many studies have been published about giving and receiving bad messages. However, only a few of them have followed the patients all the way through a disease as is done in this study. Many studies have been written about patients' coping strategies. In this study we will keep within the bounds of coping through information only. The aim of the study is to investigate patients' views of information during the trajectory of their disease, whether their reactions differ from each other and whether they differ in different phases of the disease.

**Methods:**

Twelve patients with malignant haematological diseases or lung cancer were followed with interviews from diagnosis to recovery or into the terminal phase or at most for two years. The method is qualitative, using semi-structured interviews.

Setting: Örebro University Hospital or the patient's home.

**Results:**

All patients described themselves as well informed from the start but in later phases of their disease some of them came to express a great uncertainty about the progressing disease and about the approaching death. Most of them, regardless of whether they had a haematological malignancy or lung cancer, expressed a wish to be well informed all through the disease and even when the messages were bad. Different strategies for coping with information, however, affected how they then dealt with the information received. Four such coping strategies were found: 1) Information-dependent and accepting; 2) Information-dependent but denying; 3) Medically informed and accepting; 4) Medically informed but denying.

**Conclusion:**

To several patients there was an unmet need for information about the progressing disease and the approaching death. To optimize the care of these patients it seems important that the physician is aware of patients' need for information even when the news is bad. Knowing the patient's information strategy could probably function as a key for the physician to communicate with patients on these matters.

## Background

Many studies have been published about giving and receiving bad messages. However, only a few of them have followed the patients all the way through a disease as is done in this study. Many studies have been written about patients' coping strategies since the fundamental work of Lazarus & Folkman on stress and coping [1]. In this work, however, we will confine ourselves to coping through information only, which of course is only a small part of the coping process as an entity.

Ptacek & Eberhardt [2] reviewed studies published 1985–1996, studies about the importance of where, when and how bad messages should be delivered [3-5]. Since 1996 a large number of additional studies have been published focusing on the disclosure of the diagnosis [6,7] or when death is coming close [8,9] or the impact of the social and cultural context for truth-telling [10], and that common beliefs about what a patient really wants to know could be misleading [11]. It has also been found that a majority of patients do wish to be informed even when the messages are bad [12,13].

Some studies are close to the areas of inquiry of this study, as when van der Molen [14] stresses information as a key coping strategy, Quirt et al [15] analyse how patients with recently diagnosed lung cancer received and held on to information, Leydon et al [16] and Yardly et al [17] emphasized the importance of giving moderated and individualized information. Finally, there is the longitudinal study by The et al [18] of patients with lung cancer, calling attention to information as a complicated process of interaction between the physician and the patient.

The chief contribution of this study is that, by following each patient over time, it becomes possible to notice changes, if any, in the patient's reaction to information during the disease. Secondly, this study adds partly new knowledge, compared to earlier studies, in that it focuses on and compares coping strategies of two different categories of patients: patients with malign haematological diseases and those with lung cancer. Thirdly, this study adds partly new knowledge, compared to earlier studies, in the way it focuses on patients' need for information all through the disease.

### The aim of the study

The aim of this study is to explore how patients belonging to two categories of disease relate to information given by their physicians from diagnosis to cure or until the transition to terminal care becomes obvious to the patient. The aims are to investigate patients' view of the information received:

- regarding the content

- regarding the way the information was given to them

- in the longitudinal perspective, all through the disease, and as death approaches

- to find out whether the patients' ways of reacting differ from each other, and if so, how

## Methods

The study consists of recurrent interviews with 12 seriously ill patients, 7 patients with malignant haematological diseases (acute lymphocytic leukaemia, acute myelocytic leukaemia, myeloma) and 5 patients with non-operable lung cancer diseases (adenocarcinoma and squamous cell cancer). Only 20% of patients diagnosed with acute leukaemia are estimated to be alive two years later [19]. Some patients with myeloma get an aggressive form of the disease bringing them to death within a year, while others might live with good quality of life for more than ten years [20]. Among patients with non-small-cell cancer only 3–7% are alive 5 years later, while 50% die within 7 months [21].

As the patients were diagnosed at Örebro University Hospital, Sweden, they were consecutively asked to join the study. Of 11 patients with acute leukaemia or myeloma, 10 were asked and 7 of them accepted. Of 9 patients with lung cancer, 7 were asked and 5 accepted. Exclusion criteria were if the patient was unable to give informed consent or understand the questions. The interview period was 2002–2005. The settings were either the hospital or the patient's home. The total number of interviews was 88, lasting 5–90 minutes each, all depending on the current health of the patient. The intention was neither to interview the patients as soon as possible after they had received their diagnosis nor to follow the patients during the whole processes of their dying. The first interview was held from six days to six weeks after the diagnosis had been given to the patient and the interviews came to an end as soon as the patients said that they knew that they were about to die as no more treatment was possible to stop the progress of the disease.

A summary description of the participants and the number of interviews is presented in Table 1.

**Table 1:** Study participants and number of interviews

Category of patient	Number of interviews	Sex	Age
7 Haematologic	44	3 men / 4 women	37-80 years
5 Lung cancer	44	2 men / 3 women	60-71 years

The letters A-G in the following refer to patients with haematological diseases and the letters V-Z to patients with lung cancer.

### Differences in the distribution of interviews

The intention was to interview the patients with haematological malignancies every fortnight and the patients with lung cancer as they came for treatment three times monthly, but for various reasons the number of interviews came to differ among the patients. Two patients did not get the desired effect of their treatment and died rather soon, patient B only four days after the first interview and patient V after two months. Three patients were excluded from the study, patient F because she wanted to withdraw, A for geographical reasons and D as the interview questions seemed to hurt and upset him. Four patients reached the desired state of complete remission, but three of these relapsed and then went on with a second-line treatment. Only one patient did not relapse at all during the two years that the patient was followed. Seven patients were followed into the terminal phase. For the distribution of interviews, see Table 2.

**Table 2:** The distribution of interviews

Patient	Diagnosis/Treatment 1	Treatment 2	Terminally	Totally
A	3	-	-	3
B	1	-	-	1
C	9	2	2	13
D	3	-	-	3
E	6	3	1	10
F	3	-	-	3
G	11	-	-	11
V	2	-	1	3
W	6	-	1	7
X	11	2	1	14
Y	8	2	1	11
Z	8	-	1	9
				**88**

### Ethical considerations

The patients were recruited to the study during their medical visits. As soon as the physicians found it suitable the patients were given both oral and written information about the study by their physician. Following Swedish [22] and international [23] guidelines for medical research it was stressed that participation was voluntary and the patients could withdraw whenever they wanted without any negative consequences for their treatment. The research ethics committee of Örebro University Hospital approved the study.

### Data collection

All interviews, except for those with one patient, were tape-recorded, conducted and transcribed verbatim by the first author. During the interviews with the patient who did not approve of the tape recorder, notes were taken on the basis of which the interview was reconstructed, which the patient was given the opportunity to read. The taping of another three interviews unfortunately failed, due to technical problems, but notes were taken and a reconstructed interview was read to the patient (C: 6). The other times (D: 2, X: 11) the most important questions were recapitulated in the next interviews held. Each interview consisted of 5–7 questions. The very first interview followed the interview guide, see Additional file 1. Depending on what the patient said and what happened to the patient, new questions emerged, see Additional file 2.

### Analysis

The study is qualitative, with a hermeneutic approach. The steps taken in the analysis were inspired by Kvale [24] and to some extent also by Graneheim and Lundman [25]. Firstly, all typed material was read through. Then the material not containing information and messages relevant to the purpose of this study was excluded. The remaining material was divided into three domains of investigation: the disclosure of the diagnosis and information during the first treatment, during a second treatment, and terminally. As soon as they were transcribed, all the taped interviews were listened to once again to assure conformity. Then the process of coding started, piece by piece. During the process of coding, a co-reader checked that the codes summed up the text passages. Codes with similar contents, not necessarily confirming each other, were brought together into different investigation areas. One such area was how the patient came to deal with the information received. Then four possible patterns emerged, four kinds of strategies for coping with information – the four categories.

## Results

### Patients' views of information

All the patients expressed appreciation for the straightforward way they had been informed about diagnosis, prognosis, treatment plans, possible side effects, complications, and a recurrence of the disease. One patient, though, thought that that she could have been informed this openly but in a more cautious way. "They (the physicians) just came by and told me. Was it Christmas Day? Anyhow, one of the days of Christmas ... They could have told me this more nicely, I think" (X: 1). The diagnosis was a surprise to everyone, except to patient B. This patient said: "I did understand. I did know... there is only the choice of recovery or death" (B: 1). Not all patients could give the proper name of their diseases, but they all knew it was either about a malignant haematological disease or lung cancer, and without treatment they would all have been dead within a few weeks or a couple of months. They had all been informed about the prognosis of their disease, yet, one patient made an effort to talk about the disease as if it was of no such danger to her. Then she was asked if she knew of anyone who had got the disease acute leukaemia. She said she did. They had both died (A: 2, A: 3).

However, four patients out of five with lung cancer got a sudden, remarkable – but temporary – recovery shortly after the treatment had started. After this only one of the patients, and only once more, talked about the incurableness of the disease (X: 8) but as their health started to decline they all, except patient W, were wondering about the reasons behind that (Z: 4–8, Y: 5–8, X: 8–13, V: 2).

On the other hand as treatment went on the patients came to express an adjustment of being in treatment, which made the fear less for a recurrence of the disease. Now, they thought that they knew what another treatment would be like. "If I will relapse, I will relapse... but that day, that sorrow. It is no big deal if I have to go through it once more" (C: 9). "I do understand that the cancer could come again, but I am not worried if I have to go back to the hospital. Now, I know what it would be like."(E: 6).

### Patients' views of the information – when the disease is in recurrence

For one of the patients with leukaemia the information about suddenly declining test results came abruptly and without warning (C: 10), while for another the relapse was no surprise at all. This patient had been following the slowly declining test figures. When the relapse was confirmed by a bone marrow test, it was what the patient had already suspected (E: 9).

To the patients with lung cancer the recurrence of cancer started in quite a different way. These patients were all hit by different kinds of diseases, such as thrombosis (Z), recurrent pneumonia and dyspnoea (Y, X), colds and high fever (W). This seems to have made it difficult for the patients to understand when the cancer was in progress and when not. They all came to express a great uncertainty. "It is like standing at a crossroads, not knowing which way to go" (Y: 5). Sometimes it happened that they felt really ill but all the same got good news about the tumour. "Yes, (they say) the tumour has shrunk! ... I must say I had expected a little more sad news. I was prepared, as you can see (pointing at her toilet bag), that they would keep me here" (Z: 8). Sometimes these patients did ask their physicians about the cancer. "But he couldn't tell for sure! I do hope it's not the cancer coming on again!" (X: 12). The patient was then asked whether, if it was the cancer coming back, she would have wanted to know about it or not. The answer was: "Yes I would ... I would ... but, honestly I don't think he was lying to me ... but he said he couldn't promise" (X: 12).

### Patients' views of the information – in the terminal phase

Two of the patients C and V received the information verbally from their physician that the treatment had failed and that other treatment was considered to be of no use. However, patient V had to ask to get this information. "They don't tell you very much. You have to ask..." (V: 3). Patient E seemed to have become aware of the transition to the terminal phase, not from what her physician told her but mainly because of the declining values of the test results. The transition to the terminal phase seems in no way to have been obvious to the patients with lung cancer. Even when feeling worse they were told, from time to time, that the tumour had not been growing (Z: 9, Y: 10, X: 11, W: 4). Not until the very last part of their lives did the X-rays reveal the progress of the cancer. Patient Y did not talk about his death as being close and inevitably terminal until only four days before he died (Y: 11). Patient X said that even if she might not have been given straight information about the nearness to death, she understood. "I do not think he has told me so very straight, but he held my hand and so ... so I did understand what it was all about" (X:14). Another patient declared that he was all right only three days before his death. We even talked about his 70th birthday, which was to come three months later" (W: 7).

The patients followed into terminal phase were asked if they had talked with their physicians about death. They had not. Nor had they talked about when death probably was to come. Only one had: "They say up to six months or so, if I'm lucky. Nobody knows, it might be only three" (C: 12). Patient V said: "I ask quite often how long it will take before I die, but they're not able to answer! I find that very strange. I would have appreciated if there were some kind of statistics. I suppose there are, but they don't want to tell me" (V: 3). However, even if they had not consulted their physicians, several of these patients still had an understanding of when death was probably about to come, which turned out to be rather accurate. Only one patient turned his back on the whole question. He was not in the least interested in knowing. Death would come, as death comes to everyone (W: 7).

### Patients' strategies for coping with information

Different strategies for coping with information affected how the patients came to deal with the information received. Four such coping strategies were found; Information-dependent and accepting, Information-dependent but denying, Medically informed and accepting and Medically informed but denying.

The first type is exemplified by those patients who carefully followed every test value of haemoglobin, thrombocytes, neutrophile leukocytes, bone marrow tests, X-rays, spirometries etc. Their coping strategy is called Information-dependent and accepting, and their strategy may be illustrated by quotations like: "I find everything about this disease and the treatment of it interesting" (C: 2), or "I have been asking all the time, first the nurses, and if they haven't been able to answer I have asked the doctors. I do want to know what is going on" (C: 8). One of the patients told her husband every test result, from which he made statistical diagrams so that they could closely follow every change in the values (E: 4). These patients continued to express their need for information all through the disease, even when the information was sad, and they continued to follow every test result most carefully. During second-line treatment, as in the terminal phase, these patient could, just as before, give the exact figures from the results of the tests taken (C: 10, C: 11, C: 12, E: 7, E: 9, E: 10).

Patient X illustrates the second coping strategy, Information-dependent but denying. Like patients C and E, this patient wanted information, but then held the information back. In the first interview this patient said that she was informed and that she had understood but now she tried to repress it all (X: 1). In the following interviews she often returned to the fact that she did knew, but continued by saying: "then you have to deceive yourself, you know" (X: 4). When this patient understood that death was near, the reaction was: "Well, the thing now is to handle it in a positive way" (X: 14). When asked what this could mean, the answer was: "You have to deny it" (X: 14). The patient held on to this Information-dependent but denying strategy in all phases of the disease.

The third coping strategy is called 'Medically informed and accepting'. Also these patients appreciated being informed but they came to deal with the information received in another way than did the patients categorized as Information-dependent. While the Information dependent patient followed every single test result most carefully, memorizing every value number, the Medically informed patient never took such an interest in each test taken. They reckoned that they would be informed when there was something they ought to know about. "Actually, you can't keep it all in your head. It's too much" (F: 3). "I'm not one of those who checks up the test results. They'll tell me, I suppose, if there's something I should know" (G: 11). These patients found the focusing on every test result somewhat tiring and sometimes they were criticising their doctors for being "too medical" or "too focused" on the test values figures and numbers. "Too medical ...I was unlucky meeting a real medical doctor, so to speak" (D: 3). "Yes ... it's a little too technical" (G: 6). However, of these patients only patient Z was followed into the terminal phase, so it is hard from this study to tell how well these patients held on to their strategy. What was seen in this study was that two of the patients came to change their strategy slightly to the strategy of denying. Patient G, after being told that she had been very close to death, which she had not been aware of (G:9), however, in the next interview she was back as a non-denying patient. On the other hand, patient Z became all the more denying the more her health declined.

The patient using the fourth coping strategy, called Medically informed but denying, denied the importance of the information received all through the disease. The patient approved of being informed: "I want to be informed right into the middle of my eye" (W: 1) but then he seemed to take little notice of the information received. "I need not die because of this. I could die for any reason!" (W: 5). Yet, this patient said that from the start he not only knew about the tumour in the lung, but also about the growth of metastases (W: 7). When asked about how to handle bad news he answered, as before, that he always wanted information as straight as possible. Then he was asked if there had been any bad messages in the physician's information today. "No," he answered, "the thing is that there's no need for any more treatment. It's good as it is ... and I won't have to visit the hospital until the summer is over" (Z: 6). The patient died at the beginning of August.

For the categorization of patients' strategies for coping with information, see Table 3.

**Table 3:** The categorization of the patients

Patient	1 Information- dependent and accepting	2 Information- dependent but denying	3 Medically informed and accepting	4 Medically informed but denying
A			X	
B	(X)			
C	X			
D			X	
E	X			
F			X	
G			X	
V	X			
W				X
X		X		
Y	X			
Z			X	

## Discussion

### The point of knowing the patients' strategies for coping to bad news

There is a point of knowing the patient's strategy for coping with information. But it is not in trying to classify them into any of the four categories found in this study, as there might be more strategies than those four found which could perhaps have been discovered if other patients had been included. The very point of knowing the patient's strategy for coping with information, we think, is that the coping strategy could reveal a key to the physician how to communicate with the patient in difficult matters such as information about progressing cancer and approaching death. This is especially true as we found that, irrespective of coping strategy, the need for information remained of great importance all through the disease, even when life was coming to an end. However, this is not congruent with the findings of some other studies such as the one by Leydon et al [26], where it is stated that patients vary in their attitudes towards information, with different needs at different times. There is little evidence for that in this study. We found that information for most of the patients was of great importance all through the disease, but that several patients expressed great uncertainty when life came close to an end.

The number of times participants became interviewed differed from 1–14. One might then have expected that the number of interviews could have affected the perceptions and reactions of the patients' toward their disease, making a change to the patient's strategy for coping with information. As the health of the patients was declining the attitudes to the disease of most patients were changing, but not their attitudes to information. Only on some occasions did two patients change their strategy to the strategy of denying. This might also indicate that at least some patients had the ability to find "a way out", when facts are too hard to cope with. Otherwise they all hold on to the strategy they had from start.

One might also have expected that patients with lung cancer diseases and haematological diseases would have reacted somewhat differently to the information given, revealing differences in when and how much patients were informed. But we found people who were well informed and not so well informed in both kinds of patients, though the less well aware patients were more often found among patients with lung cancer. The reasons behind that need to be investigated more.

The age of the interviewees varied, from 37–80 years. The age could then have been of importance for the strategy of the patient's but this was not found. The youngest patients were categorized differently in relation to each other, as were the oldest patients.

Finally one might have expected gender differences among the patients' attitudes to information but no such differences were found.

The finding that patients were well informed from the start but not in later phases of their disease has been observed in the literature though different explanations are given, such as that it is due not only to the information given by the physicians, but also to the patient's ability to understand the verbal utterances of the physician, as well as to the patient's ability to cope with and integrate the bad news into his or her life [27]. More research is needed to find out whether the deficiency is due to a lack of information by doctors or to a lack of knowledge by patients to understand the information provided.

### How to deal with the lack of knowledge among patients?

The answer to this question will obviously depend on the reasons behind the lack. Some patients might need further information, others might need to have the information explained, or repeated. Still other patients might need to cope with the information by leaving it behind them. Improved disclosure and the patients' right to obtain information about their condition can be supported by the principle of autonomy [28]. Non-disclosure can be supported by the principle of beneficence [29]. In the study by Schapira [30] the tension between those who favour complete disclosure and those who prefer more limited truth-telling is confirmed. Both practices, she says, can be justified ethically and both have received support in the published literature. We believe that the ethical justification is an act of balancing the pros and cons for what seems to be the best for the patient. But it has to be emphasized what is found in many studies and confirmed in this one is that most patients want to be well informed even when the messages are bad.

To optimize care of patients it seems important that, irrespective of the reason behind the lack of knowledge among patients, it is recognized and as much as possible minimized by their physician. We believe that a patient with a better knowledge about approaching death is more able to make the necessary and desired practical, economic, social and spiritual adjustments and preparations in time before his or her death. Talking about death could probably harm some patients, at least in the short term, but if patients are as information-dependent as shown in this study and if the physicians are good at telling even sad news, as reflected in the interviews of this study, we are convinced that most patients would be relieved.

### Limitations, trustworthiness and validity

In this study the first author had the unique opportunity to follow two groups of patients with severe disease over time. The patients, however, were not interviewed during the first shocking phase after having received their diagnosis. This might have affected the patients, so that they had a more positive attitude from the start, that is, to the information received, to the way it was given to them and to the informing physician.

As already noted only seven out of twelve patients, for various reasons, were followed into the terminal phase (see Table 2).

The intention to maintain some regularity in following the patients could not always be realized. Some prearranged interviews had to be shorted in time or postponed for days, or even weeks, because of the patient's actual state of health.

A final important point is that not all patients' strategies for coping with information were easy to identify. The patients in categories 1 and 4 were easy to identify, but not patients in categories 2 and 3. How to categorize these patients was very much a question of what to emphasize. By balancing the patients from one category to another, it became obvious where the patients fitted best.

Of course, the interview material can also be criticized for not being reproducible, as each interview reflects what the patients remembered about the past in a fleeting moment when they were met [31]. However, each interview is a valuable source of information even if the results cannot be generalized. The methodology is chosen to be appropriate for the questions formulated [32]. The intention was to follow as closely as possible the words of the patients, but there is always the possibility of misinterpretations or over-interpretations. To minimize such risks most interviews started with a short recapitulation of the previous one held. Checking questions were also used (dialogic validity) [33]. Finally, all through the study, critical researchers, physicians and co-readers have critically read and checked the interpretations made (communicative validity) [34].

## Conclusion

The result of this study could help to improve health care, if it could make physicians

- more aware of the fact that there are patients whose need for information remains of great importance all through the disease, even in later phases and when life comes near to the end

- more aware that there is sometimes a lack of knowledge among patients, to be recognized and as much as possible minimized

- more aware that knowing the patient's strategy for coping with information could probably function as a key for the physician when to communicate with the patient about progressing disease and about death coming near

## Competing interests

The author(s) declare that they have no competing interests.

## Authors' contributions

UT has contributed to the conception and design of the study and was the medical supervisor concerning patients with haematological diseases and responsible for the selection criteria. LT was the medical supervisor concerning the patients with lung cancer and responsible for the selection criteria. GH has contributed to the conception and design of the study and was the philosophical and ethical supervisor. They have all given substantial criticism during the process of writing by LH, who was the interviewer, data collector, analyser and writer. All authors have read and approved the final manuscript.

## Pre-publication history

The pre-publication history for this paper can be accessed here:



## Supplementary Material

Additional File 1 Appendix 1. Interview guide (to the first interview with each patient) Click here for file

Additional File 2 Appendix 2. Interview guide (to remaining interviews) Click here for file
