# Supplementary material for: In the shadow of bad news – views of patients with acute leukaemia, myeloma or lung cancer about information, from diagnosis to cure or death
Source: BMC Palliat Care. 2007 Jan 24;6:1. doi: 10.1186/1472-684X-6-1 (PMC1794231; doi:10.1186/1472-684X-6-1)
Supplement: Additional File 1 — Appendix 1. Interview guide (to the first interview with each patient) [file 1472-684X-6-1-S1.doc]

**Appendix 1 - Interview guide (to the first interview with each patient):**

1) I don’t know much more about you other than the fact that you are seriously ill. Do you know the name of the disease that you have? What made you visit the doctor? What symptoms did you have?

2) From the kind of symptoms you told me about, I think it must be hard to believe in the seriousness of the disease. You went to the doctor, “almost” healthy but left him/her very ill. Was this surprising for you? Or were you suspecting the diagnosis could be bad, and that bad?

3) How are you now? What are the doctors telling you about the future? Is there anything said about the prognosis for you?

4) What kind of treatment are you getting? Do you know? Did you and your physician discuss any alternatives? What do you say about the treatment plan for you? What does the treatment plan mean to you? Did you immediately approve of it?

5) Do you know how long it will take until you know whether the treatment will be effective?
