# Supplementary material for: In the shadow of bad news – views of patients with acute leukaemia, myeloma or lung cancer about information, from diagnosis to cure or death
Source: BMC Palliat Care. 2007 Jan 24;6:1. doi: 10.1186/1472-684X-6-1 (PMC1794231; doi:10.1186/1472-684X-6-1)
Supplement: Additional File 2 — Appendix 2. Interview guide (to remaining interviews) [file 1472-684X-6-1-S2.doc]

**Appendix 2 - Interview guide (to remaining interviews)**

First a short recapitulation of the previous interview and then questions like:

1) What has happened to you since we last met?

2) How are you today? What is worse? What is better?

3) What is there to tell about the treatment you are getting?

4) Any news in the information given to you?

5) What now?
